# The Androgen Receptor in Prostate Cancer: Effect of Structure, Ligands and Spliced Variants on Therapy

**DOI:** 10.3390/biomedicines8100422

**Published:** 2020-10-15

**Authors:** Elisabeth A. Messner, Thomas M. Steele, Maria Malvina Tsamouri, Nazila Hejazi, Allen C. Gao, Maria Mudryj, Paramita M. Ghosh

**Affiliations:** 1 VA Northern California Health Care System, Mather, CA 95655, USA; eamessner@ucdavis.edu (E.A.M.); tmsteele@ucdavis.edu (T.M.S.); mtsamouri@ucdavis.edu (M.M.T.); acgao@ucdavis.edu (A.C.G.); mmudryj@ucdavis.edu (M.M.); 2 Graduate Group for Integrative Pathobiology, University of California Davis, Davis, CA 95616, USA; 3 Department of Urologic Surgery, University of California Davis, Davis, CA 95718, USA; 4 Department of Clinical Science, California Northstate University, Elk Grove, CA 95757, USA; nazila.hejazi@cnsu.edu; 5 Department of Medical Microbiology and Immunology, University of California Davis, Davis, CA 95616, USA; 6 Department of Biochemistry and Molecular Medicine, University of California Davis, Davis, CA 95718, USA

**Keywords:** castration-resistant prostate cancer, androgen receptor, N-terminal domain, DNA-binding domain, Ligand-binding domain, alternatively spliced variants

## Abstract

The androgen receptor (AR) plays a predominant role in prostate cancer (PCa) pathology. It consists of an N-terminal domain (NTD), a DNA-binding domain (DBD), a hinge region (HR), and a ligand-binding domain (LBD) that binds androgens, including testosterone (T) and dihydrotestosterone (DHT). Ligand binding at the LBD promotes AR dimerization and translocation to the nucleus where the DBD binds target DNA. In PCa, AR signaling is perturbed by excessive androgen synthesis, AR amplification, mutation, or the formation of AR alternatively spliced variants (AR-V) that lack the LBD. Current therapies for advanced PCa include androgen synthesis inhibitors that suppress T and/or DHT synthesis, and AR inhibitors that prevent ligand binding at the LBD. However, AR mutations and AR-Vs render LBD-specific therapeutics ineffective. The DBD and NTD are novel targets for inhibition as both perform necessary roles in AR transcriptional activity and are less susceptible to AR alternative splicing compared to the LBD. DBD and NTD inhibition can potentially extend patient survival, improve quality of life, and overcome predominant mechanisms of resistance to current therapies. This review discusses various small molecule and other inhibitors developed against the DBD and NTD—and the current state of the available compounds in clinical development.

## 1. Introduction

**Figure 1:**
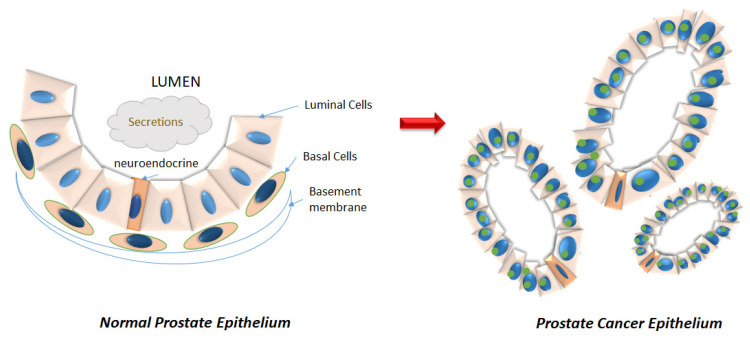
Disruption of the prostatic epithelium during neoplastic progression. (**Left**) The normal prostatic acinus consists of an epithelial compartment consisting of basal (orange) and luminal (pink) cells, as well as a minor population of neuroendocrine cells (darker orange) that serve as stem cells in case of damage repair. The basal cells line the basement membrane. (**Right**) Cancer is characterized by luminal hyperproliferation resulting in the formation of multiple new glands, loss of basal cells, breakdown of basement membrane, prominent nucleoli (green), and nuclear enlargement.

The prostate gland, located beneath the bladder, secretes alkaline prostatic fluid that constitutes 30% of the semen [1]. The prostate is composed of three distinct zones: the central zone (CZ), which includes the ductal tube from the seminal vesicle to the descending urethra, the peripheral zone (PZ), which is situated at the posterior of the gland and the transitional zone (TZ), which surrounds the transitional urethra [1]. The normal prostatic acinus consists of an epithelial structure of basal and luminal cells surrounded by fibromuscular stroma (Figure 1). Luminal, basal, and neuroendocrine cells constitute the normal prostate epithelia in a roughly 60:40:1 ratio [2]. Prostate cancer (PCa) develops most often in the PZ, less often in the TZ, and rarely in the CZ [3,4]. Upon development of PCa, the ratio of luminal to basal percentages are greatly altered, with the luminal cells constituting >99% of the tumor [2] (Figure 1).

Among men worldwide and in the United States, PCa is a leading contributor to overall cancer incidence [5]. In 2020, 191,930 new cases and 33,330 deaths are predicted for PCa in the United States [5]. The mean age of initial diagnosis is 66 [6]. According to the Surveillance, Epidemiology, and End Results (SEER) Cancer Stat Facts: Prostate Cancer Survival Statistics, which reports survival by stage, the relative 5-year survival rate for PCa initially diagnosed when localized (within the prostate gland), or regional (external to the prostate in adjacent lymph nodes and seminal vesicles), is almost 100% [7,8]. However, for patients with distant metastasis at initial diagnosis, the relative 5-year survival drops to 30.2% [7,8]. Despite diagnostic advancements in the past 30 years, only about 76% of diseases are initially diagnosed when they are still localized [9]. About 13% of the patients are first diagnosed with regional disease, while 6% are diagnosed with distant metastasis (5% remain unstaged) [7,8].

Serum levels of prostate-specific antigen (PSA), a serine protease produced in the prostate epithelium, can be used both to diagnose and to track PCa. While the diagnostic capability of PSA is limited, it remains the main biomarker of PCa progression. The androgen receptor (AR), the main promoter of PCa, regulates *PSA* gene transcription, making serum PSA levels a strong indicator of AR transcriptional activity and subsequently of disease state. Specifically, PSA levels are positively associated with disease stage and clinical grade [10]. PSA takes on a particularly significant role in PCa to indicate treatment efficacy and relapse [10]. Thus, a reduction in PSA following treatment is often interpreted as the abatement of disease. Retention in circulation following PCa treatment indicates the continued presence of prostate-derived cancer in the system [10].

Three possible treatments for localized PCa are expectant management, surgery, and radiation. Expectant management, for low-risk disease, includes either watchful waiting or active surveillance [11]. Radical prostatectomy and/or radiation may be performed in cases of a more advanced localized cancer [12]. However, 20–40% of patients who undergo these treatments will experience biochemical recurrence (BCR), involving increasing PSA levels marking disease progression [13]. For patients with BCR or with metastatic PCa, androgen deprivation therapy (ADT), a treatment aiming to reduce cancer growth and alleviate pain by reducing circulating androgens, becomes the standard of care treatment [9,12,13,14]. Within 2–3 years, patients often develop resistance to ADT monotherapy, resulting in castration-resistant PCa (CRPC) [9,15,16,17,18,19].

## 2. Structure of the Androgen Receptor

**Figure 2:**
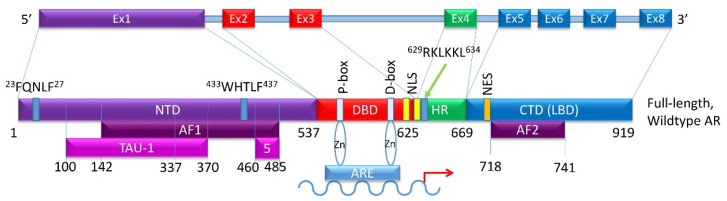
Schematic of the canonical full-length wild type AR exons and the domains they encode. (**Top**) Exon structure of the *AR* gene. (**Bottom**) Protein domains of the full-length wild-type AR showing which exon encodes for which domain. Additional minor domains are also indicated. The P-box and the D-box identify two zinc-fingers that directly find to the ARE on the target gene.

The 90 kb *AR* gene is located on Xq11-12 and encodes a 110 kDa, 920 amino acid protein that consists of a N-terminal domain (NTD), a DNA-binding domain (DBD), a hinge region (HR), and a C-terminal region that contains the ligand-binding domain (LBD) (Figure 2) [20]. The full-length monomeric canonical 110 kDa AR contains eight exons [9,14] (Figure 2); of these, exon 1 encodes the NTD, exons 2–3 encode the DBD, exon 4 encodes the HR, while exons 5–8 encode the LBD [14,21,22,23]. As yet, no crystal structure for the full-length AR exists; however, the structures of the AR LBD and, separately, the AR DBD when bound to DNA, have been resolved [21,24,25].

The NTD has been reported as a significant activation domain for the AR because of its necessary presence for LBD activation. Within this domain, activation function 1 (AF1) located between amino acids 142 and 485, is the main region responsible for mediating AR transcription (Figure 2). The NTD contains two additional transcriptional activation units, Tau-1 (between amino acids 100–370) and Tau-5 (between amino acids 360–528) [26]. Tau-1 has been reported to be dependent on LBD function and can be bound by co-activators and co-repressors that help regulate AR transcriptional activity [26]. Under normal conditions, interaction between the NTD and LBD is required for AR transcriptional activity [27]. The ^23^FQNLF^27^ motif at the N-terminal interacts both with activation function 2 (AF2) on the C-terminus and with coactivators in a ligand-dependent manner to form an AR dimer (Figure 2). Additionally, the complex between the AR and androgen ligand is stabilized by an interaction between a ^433^WHTLF^437^ motif in the NTD and regions of the LBD [28]. Co-regulators can bind to the NTD to influence AR localization, ARE binding, and transcriptional activity [29].

The DBD is critical for AR function. It plays a role in AR dimerization and binding of dimerized AR to select motifs on target DNA [30]. Contributing to these DBD functions are cysteine residues located within this domain that facilitate the formation of two zinc finger motifs. The first is closer to the NTD, has the P box, and controls the DNA binding specificity at specific DNA sequences (Figure 2). These sequences are typically identified as androgen response elements (AREs), cis-acting sequences located in the regulatory regions of genes [31]. The second zinc finger motif facilitates AR dimerization via the D box [32]. Additionally, a nuclear localization signal (NLS) shared with the HR is located partially in the DBD (amino acids 608–625) and is responsible for nuclear translocation of androgen-bound AR [33]. 

The hinge region, located between the DBD and LBD, is a flexible linker that is poorly conserved. The hinge region shares an NLS with the DBD that binds to importin-α and facilitates nuclear translocation [34]. The ^629^RKLKKL^634^motif of the hinge region allows nuclear translocation through possible interactions with nuclear import machinery (Figure 2). Once in the nucleus, the hinge region also interacts with the DBD to identify specific sequences for AR binding. The hinge region controls the potency of AR activation and mediates AR degradation. Consequently, mutations in the hinge region can lead to enhanced AR potency [35].

The LBD, located at the C-terminus, is the main target of AR inhibitors. This region consists of 11 β-helices in the ligand binding pocket, which reposition upon androgen binding, converting into the activation function 2 (AF2) domain (amino acids 718–741) (Figure 2). The LBD binds FXXLF motifs in the NTD and in AR-specific cofactors. It can also bind some LXXLL motifs of coactivators [36]. LBD-LBD homodimerization of AR induced by receptor agonists, such as activating ligands, is an essential step in the proper functioning of the AR [24]. A number of mutations in the LBD have been identified in PCa. It is important to note that not all AR-LBD mutations affect ligand binding. Some mutations may disrupt androgen induced interaction of the N-terminal motif FXXLF and the C-terminal activation function 2 (AF2) [37]. Thus, each domain of the AR aids in controlling and regulating transcriptional activity.

## 3. The Spaciotemporal Regulation of AR Transcriptional Activity

**Figure 3:**
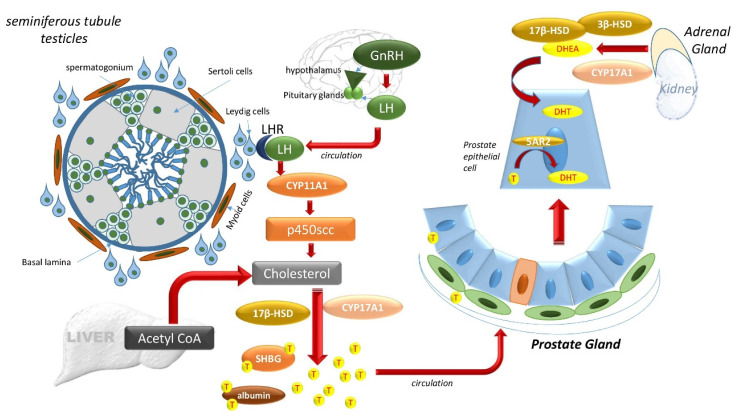
The hypothalamus-pituitary-testes axis and the adrenal gland controls DHT. The hypothalamus produces gonadotropin-releasing hormone (GnRH) to stimulate luteinizing hormone (LH) release by the anterior pituitary system. LH travels via circulation to the testes where it binds to LH receptors on Leydig cells outside of the seminiferous tubules of the testicles and regulates the activity of P450scc (encoded by *CYP11A1*), a member of the cytochrome P450 superfamily of enzymes. The substrate of P450scc is cholesterol, and multiple enzymatic reactions result in T synthesis via 17β-hydroxysteroid dehydrogenase (17β-HSD), also known as aldo-keto reductase family 1, member C3 (AKR1C3). Newly synthesized T is then secreted into the circulation, which carries it to the prostate. In the prostate cells, T may be converted via 5α-reductase (5AR) into DHT, which can also be produced from adrenal dehydroepiandrosterone (DHEA) via 3β-HSD and Cytochrome P450 17α-hydroxylase/17,20-lyase (*CYP17A1*).

Two main ligands that bind and activate the AR at the LBD are testosterone (T) and its metabolite dihydrotestosterone (DHT) [38]. The hypothalamus-pituitary-testes axis controls the majority of T synthesis (Figure 3). The hypothalamus produces gonadotropin-releasing hormone (GnRH) to stimulate luteinizing hormone (LH) release by the anterior pituitary. LH travels via circulation to the testes where it binds to LH receptors on Leydig cells and regulates the activity of P450scc (encoded by *CYP11A1*), a rate-limiting enzyme that begins T production from cholesterol [32]. Multiple enzymatic reactions then result in T synthesis via 17β-hydroxysteroid dehydrogenase (17β-HSD) (Figure 3). Newly synthesized T is then secreted into the circulation. About 90% of T in circulation is produced in the Leydig cells of the testes, whereas the rest is supplied by the adrenal cortex. Most circulating T binds to either sex hormone-binding globulin (SHBG) or to albumin (Figure 3). A negative feedback loop prevents further hypothalamus release of LH [21,32].

Once internalized by prostate cells, T may be converted via 5α-reductase (5AR) into its higher affinity form, DHT. Two isoforms of 5AR are known, 5AR1 and 5AR2, which differ in their T affinity. 5AR1 exhibits a low affinity for T and can be expressed in non-prostatic tissue like the liver, skin, and hair follicles. 5AR2, with a high affinity for T, is mainly expressed in prostate tissue. In normal prostate tissue, basal epithelial cells express 5AR1 in the nuclei, while epithelial and stromal cells express 5AR2 in the cytoplasm [39]. Although both T and DHT activate AR, AR has a two- to five-fold higher binding affinity for DHT than T, making DHT the more effective intraprostatic androgen [32,38].

**Figure 4:**
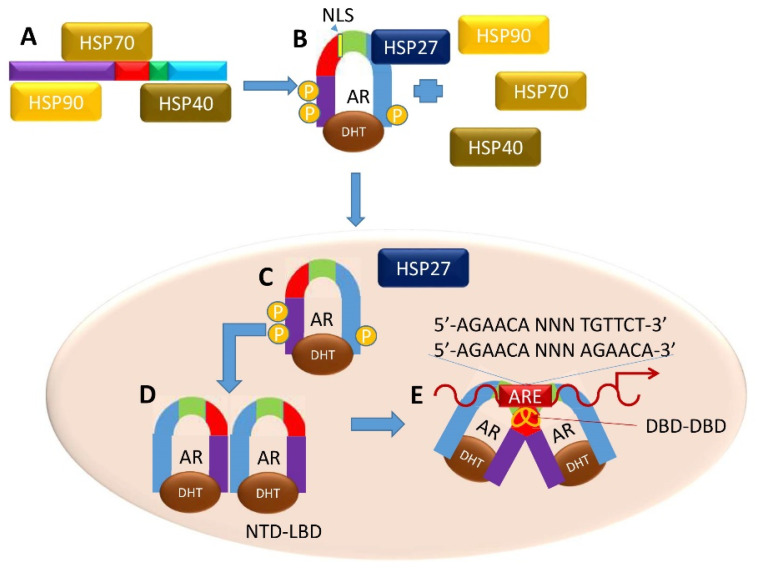
Canonical Genomic AR Signaling Pathway. (**A**) Upon androgen binding to AR, heat-shock proteins dissociate and (**B**) the AR forms NTD-CTD monomers. (**C**) These monomers translocate to the nucleus, and (**D**) form NTD-LBD homodimers. (**E**) Both androgen-bound homodimers (**C**,**D**) form a DNA-dependent DBD–DBD homodimer at AREs to enact a transcriptional program related to normal prostatic function.

In the normal prostate, as T is internalized by the cell, the AR can be transcriptionally activated with ligand binding. In the cytoplasm, the inactive, unliganded, full-length AR monomer is bound to heat shock proteins (HSP) 90, 70, and 40 of the HSP chaperone complex, protecting the AR from degradation (Figure 4A) [40]. Upon androgen binding at the LBD, the monomeric AR undergoes a conformational change, allowing its dissociation from HSP-90, -70 and -40 and association with HSP27 [9,14,15,21,31,41,42,43]. One model suggests this conformational change prompts phosphorylation of the AR resulting in an intramolecular interaction between the NTD and LBD (Figure 4B), exposing the nuclear localization signal and allowing subsequent entry into the nucleus through the nuclear pore complex (Figure 4C) [24,44].

In the nucleus, new intermolecular interactions arise, and subsequent steps allow intermolecular interactions to occur between the DBDs of two ligand-bound AR monomers. The D-box regions bind to the partner monomer (DBD–DBD interaction), and the P-box regions of each AR monomer binds to the target DNA (Figure 4D) [42]. The DBD–DBD “head-to-head” homodimer (Figure 4E) [23,42,44] forms at AREs defined either by the canonical two hexameric palindromic half-site sequence 5′-AGAACA-NNN-TGTTCT-3′, or by the AR-specific two hexameric direct repeat sequence 5′-AGAACA-NNN-AGAACA-3′ [25,31]. LBD-LBD homodimers (not shown) may also form in an androgen-dependent manner, but the spaciotemporal regulation of this dimer is yet to be uncovered. AR also binds to non-canonical AREs, although it is unclear the extent to which non-canonical AR binding is functional [45]. Upon DNA binding, the AR homodimers become receptive at the NTD to co-regulatory proteins that help promote or inhibit gene transcription [46].

Co-regulators activate (co-activator) or repress (co-repressor) transcription by altering the 3D structure of chromatin, influencing the stability of the receptor, facilitating nuclear transportation, affecting DNA binding, and regulating molecular interactions [32,47]. In CRPC, changing levels of AR co-activators and co-repressors may augment its activity [48]. ARA70 and filamin A are co-activators that stabilize androgen-bound receptor and facilitate nuclear translocation, respectively [32,47,49,50,51,52,53]. Likewise, steroid receptor coactivator 1 (SRC1) and human transcriptional intermediary factor 2 (TIF2), both members of the p160 coactivator family, interact with AF2 to control AR activity [35,54]. Other transcription factors (TFs) shown to modulate AR transcriptional activity include the ETS family [55], GATA, FOXA1 [56,57], nuclear factor B (NFB), sex-determining region Y (SRY), and Smad3 [57]. Calreticulin, a co-repressor, impedes DNA binding and inhibits AR transactivation by interacting with the AR DBD [32,47], and the co-repressor FOXO1 directly interacts with the AR NTD to inhibit co-activator interaction [29]. Together, coregulatory proteins define the AR transcriptional program.

Post-translational modifications influence both AR genomic activity and non-genomic protein dynamics in the cytoplasm or nucleus. The AR can acquire modifications throughout all domains, with phosphorylation and SUMOylation sites concentrated in the NTD, acetylation and methylation sites in the DBD, and ubiquitination sites localized mostly to the LBD [58]. Such modifications may control AR-protein dynamics, co-regulator recruitment, gene transcription repertoire, AR activity, or AR protein stability [58]. These mechanisms work cohesively to regulate AR function, stability, structure, and activity to maintain homeostasis within a normal tissue ecosystem. Nonetheless, in oncogenesis and established cancer, these mechanisms either become altered or subverted to allow tumorigenic AR activity.

In the normal environment, production of T and conversion to DHT regulate AR activation through the LBD. Co-regulatory proteins and post-translational modifications aid in defining the AR transcriptional repertoire and tuning AR activity. In PCa, these pathways may become dysregulated, subverted, or hijacked to cause outlaw AR activation and the production of an oncogenic transcriptional program. This implicates the AR as a major promoter of cancer growth and disease progression. Indeed, the genes most often altered in hormone-naïve PCa, which is characterized by no prior treatment with hormone therapies, are those involved in the AR signaling axis [14]. Often, a functional AR remains a key regulator of disease, and its activity is no longer dependent on androgens [14,59].

## 4. AR-Driven Resistance to Current Treatments for Advanced and Metastatic CRPC

In a 1941 landmark study, prominent Urologists Drs. Charles Huggins and Clarence Hodges reported on the benefits of castration to aid in the reduction of advanced and metastatic PCa burden and the worsening of clinical condition when androgens were administered. This study highlighted the importance of androgen in driving disease state. Based on these observations, Dr. Huggins received a Nobel Prize in Physiology or Medicine in 1966 “*for his discoveries concerning hormonal treatment of prostatic cancer*” [17,60,61,62]. Today, ADT, a form of “*castration*”, remains key to the treatment of advanced and metastatic PCa. ADT is achieved through surgical, or more commonly chemical, castration [14]. ADT starves the AR of androgen to successfully prevent pro-tumor transcriptional activity, emphasizing the initial importance of the AR in PCa pathology. Androgen sensitive disease is defined by ADT efficacy where many of the oncogenic cells undergo apoptosis and the surviving cells are arrested in the G1 phase [48].

ADT frequently uses GnRH agonists that prevent androgen production [63,64,65]. Commonly used drugs of this class include leuprolide acetate, goserelin acetate, triptorelin, and histrelin. GnRH agonists initially, but briefly, increase the production of androgens in the testes [15,61]. Despite this “flare,” the continuous stimulation of pituitary GnRH receptors causes the eventual downregulation of androgens and desensitization of the AR. This halts LH release and achieves an overall reduction in circulating T, estrogen, and progesterone [61]. In cases when the T flare is undesirable, GnRH antagonists are also used. The latter function differently, but they achieve the same goal of T reduction. GnRH antagonists reversibly inhibit GnRH receptors on the anterior pituitary gland, preventing LH secretion [62]. To ensure full blockade of T production, adrenal androgen inhibitors, including corticosteroids, ketoconazole, and aminoglutethimide, may also be used [61], though the toxicities of ketoconazole often outweigh its benefits [17]. Regardless of the form of ADT, however, within 2–3 years, most PCa patients develop resistance to ADT monotherapy and progress to CRPC [9,15,16,17,18,19]. In addition to the above, competitive inhibitors of ligand binding to the AR-LBD, the first generation antiandrogens such as bicalutamide, nilutamide, and flutamide, were also developed; these anti-androgens allow the AR to enter the nucleus and bind to target DNA but recruits co-repressors [66] and repels co-activators [66,67] to halt AR transcriptional activity.

During CRPC progression, subversions within the cell allow a continued oncogenic transcriptional program and a rise in serum PSA despite low serum androgen levels [9,14,15,21,41]. Mechanisms of resistance to ADT may be both ligand-dependent and independent—ligand-dependent mechanisms include the amplification of AR gene and/or protein expression, new or increasing tumor cell steroidogenesis, increased cellular uptake of androgens, AR receptor promiscuity, and increased expression of 5α-reductase [9,14,15,41]. These mechanisms of resistance result in excessive production of either the AR itself or of its ligand(s) that keep the LBD active and engaged. The resulting increase in AR transcriptional activity cannot be curtailed by ADT monotherapy.

Additionally, in CRPC, increased androgen synthesis may occur due to upregulated key enzymes used in the steroidogenesis pathway. Upregulation of enzymes that convert androstenedione to T, including aldo-keto reductase family 1, member C3 (AKR1C3), also known as 17β-HSD, may lead to elevated androgen levels in this context [68]. In these patients, both mRNA and protein levels of AKR1C3 are increased [38]. 3β-HSD, the enzyme that converts DHEA to androstenedione, is also frequently upregulated in CRPC. Following ADT, progesterone levels may be increased in CRPC tumors. Because progesterone is an androgen synthesis precursor, the increased progesterone may be important for de novo synthesis of DHT in CRPC [69]. Similar to progesterone, increased levels of DHEA and androstenediol are found in CRPC tumors and significantly contribute to de novo DHT synthesis. Whether these androgens come from circulation or are synthesized within the PCa cells remains unclear [38]. The combination of increased levels of AKR1C3, 3β-HSD, progesterone, DHEA, and androstenediol may produce de novo DHT in CRPC [68]. Notably, 5AR1 and 5AR2 both increase following ADT. 5AR1 expression and enzymatic activity are considerably increased in CRPC. Increased levels of androgen precursors and enzymes in steroidogenesis strengthen the hypothesis that CRPC develop intratumoral androgen synthesis as compensatory mechanisms of resistance [39,70].

AR mutations can also function as a ligand-dependent mechanism of resistance, because in many cases, mutant AR can bind to ligands other than androgens in order to be activated [23]. Additionally, mutant AR may be activated by castrate levels of androgens. These mutations are likely responsible for continued AR activation despite castrate levels of androgens in the serum following ADT and transition into CRPC, where 15–40% of original DHT levels is sufficient to stimulate AR signaling [39]. Mutations may result in reduced ligand-binding specificity and/or AR activation by estrogens, progestin, adrenal androgens, glucocorticoids and AR antagonists [71], rendering ADT ineffective.

While ADT inhibits production of testicular testosterone, it does not impact production of adrenal DHEA or intratumoral androgen, which can also drive CRPC. The *CYP17A1* inhibitor abiraterone acetate (ABI) prevents steroidogenesis and subsequently halts, although sometimes incompletely, adrenal production of T [9,17]. Initially approved in 2011 for post-docetaxel CRPC [72], the use of ABI in combination with prednisone has since expanded to first line therapy for newly diagnosed CRPC [73], and more recently, for use in patients with castration sensitive PCa [74]. Patients who best respond to ABI have activated full-length AR and suffer from disease progression caused by ligand-dependent mechanisms of resistance delineated above [75].

In addition to ABI, AR inhibitors have been developed and investigated as both first- and second-line therapies to address some mechanisms of resistance utilized by CRPC [17,19]. Next generation non-steroidal antiandrogens like ENZA (now approved for first line therapy against CRPC as well as castration sensitive PCa [76]), darolutamide, and apalutamide function in a mechanism similar to the first-generation anti-androgens but act more potently [9,17,19]. ENZA inhibits AR activity by obstructing androgen-AR interaction, preventing AR nuclear translocation and thus AR-DNA binding, and impeding co-activator recruitment [9]. Apalutamide binds the same site as bicalutamide but exhibits a 7- to 10-fold greater affinity for the AR to efficiently inhibit androgen binding [77]. The AR antagonist darolutamide has recently been approved by the FDA for the treatment of non-metastatic CRPC [78]. This new therapy may have fewer side effects compared to ENZA and apalutamide due to its distinct chemical structure and lower penetration of the blood-brain barrier [78].

Ligand-independent resistance mechanisms can result from altered co-regulator repertoire that modulates the various steps of AR activation, increased growth factor-AR crosstalk, altered post-translational modifications to AR, and alternative splicing of AR mRNA [9]. Certain AR mutations may result in ligand-independent activation of the receptor [79]. AR mutations develop throughout PCa progression and vary depending on the extent of metastasis, and/or administration of ADT and other therapies [48]. Studies have reported over 660 mutations in AR with a <25% frequency of mutation in androgen-dependent PCa and a >50% frequency in androgen-independent and metastatic PCa [80]. Combining the two groups, 40% of mutations occur in the LBD, 37% of mutations are located in the NTD, and 9% of mutations are found in the DBD [80]. Most AR mutations are gain-of-function point mutations [81]. For example, the T877A mutation in the LNCaP and LNCaP-derived cell lines allows bicalutamide to promote, instead of inhibit, AR genomic activity [21].

## 5. AR Variants in the Development of Resistance to Current AR Inhibition Strategies in CRPC

**Figure 5:**
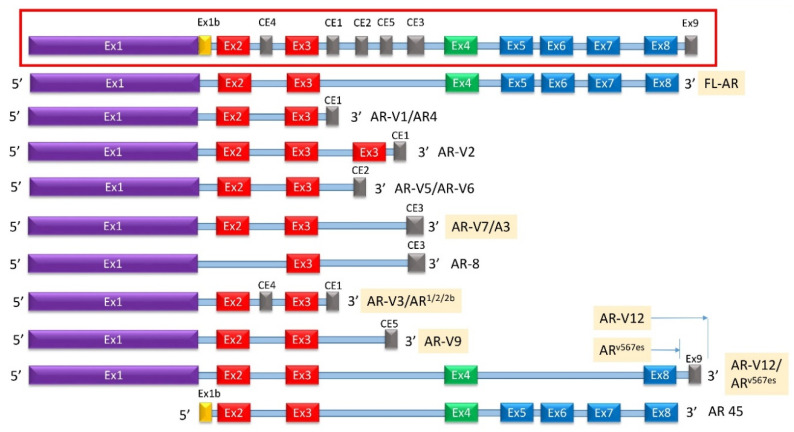
Known AR-Vs Lacking Specific Domains. (**Inset**) A simplified schematic of the gene encoding for AR with designated cryptic and canonical exons as well as intronic sequences known to be integrated into alternatively spliced AR protein products. (**Main**) The subset of AR-Vs known to lack the LBD are shown above with the translated sequences represented on the left and the name(s) of the correlating variant on the right. All known AR-Vs lacking the LBD do contain the full NTD and at least one exon of the canonical DBD. Of note, AR-V5 and AR-V6 are distinct variants that contain different translated 3′ sequences of CE2. CE: cryptic exon, CTD: C-terminal domain, DBD: DNA-binding domain, Ex: Exon, HR: Hinge region, LBD: Ligand-binding domain, NTD: N-terminal domain.

AR alternative splicing produces AR variants (AR-Vs) that are contributors to ligand- and LBD- specific AR treatment resistance. Many known, and most clinically relevant, AR-Vs lack the LBD, making them naturally resistant to ADT (Figure 5), LBD-specific anti-androgens, androgen synthesis inhibitors and AR-LBD inhibitors. AR-V presence in PCa often leads to aggressive disease and a worsened clinical prognosis [9,22,43]. Indeed, variant gene signatures have been associated with disease progression with respect to histological grade, metastases, and BCR [82]. These variants may also have a role in eliciting resistance to taxanes in CRPC [83]. Mechanistically, ADT and AR signaling inhibitors (ASI) that include both androgen synthesis inhibitors, like ABI and AR inhibitors, like ENZA, select and enrich for AR-V ligand-independent modes of action [43,84,85]. ENZA and ABI resistance corresponds to AR-V presence in circulating tumor cells of patients with CRPC [83].

AR-Vs can dimerize with each other to work independent of the full-length AR, or they can dimerize and work synergistically with the full-length AR [22,83]. This process may occur in a ligand-independent manner whereupon the DBDs interact to form three possible dimers: AR-V/full-length AR heterodimers, AR-V/AR-V heterodimers, or AR-V/AR-V homodimers [83]. Once in the nucleus, the dimers bind DNA and promote the transcription of both a unique repertoire of genes not regulated by the full-length AR and the canonical set of AR-regulated genes [82]. Heterodimerization of AR-V and AR-FL was mediated by N/C-terminal interactions and by the DNA-binding domain of each molecule, whereas AR-V homodimerization was mediated only by DNA-binding domain interactions [86]. AR-Vs that lack the LBD and have an intact DBD are therefore likely promoters of therapy resistance; however, multiple studies have indicated that of these, the greatest effect comes from AR-V7 [87,88]. Further, depending on whether the variants express a NLS or can bind to a molecule that expresses a NLS, AR-Vs localize to either the cytoplasm or the nucleus. One known variant, AR8, localizes to the plasma membrane [83]. Constitutively active AR-Vs mostly remain in the nucleus even in the absence of androgen, while inactive AR-Vs localize to the cytoplasm unless trafficked into the nucleus by either an activated full-length AR or a nuclear-localized AR-V [83].

At least 22 AR-Vs have been described (some of which are described in Figure 5) with AR-V3, AR-V7/AR3, AR-V9, and AR^V567es^ currently clinically identifiable from blood or tissue samples associated with CRPC [83,89,90,91]. Alternative splicing results in multiple transcript variants encoding different isoforms that may involve exon skipping, exon 3 duplication, and/or cryptic exons located within introns 2 and 3 [43,91,92,93]. At least six cryptic exons (cryptic exon 1 (CE1), CE2, CE3, CE4, CE5, and a so-called “exon 9”) can be incorporated into the final transcript (Figure 5) [22]. The exons most commonly excluded are exons 5–7, followed by exon 4 and/or exon 8 [9,43]. Some variants have been identified that exclude exon 1 [94] (Figure 5). Multiple variants such as AR-2 and AR-4 exhibit exon 3 duplication [92]. 

AR-V7, also known as AR3, is encoded by splicing of exons 1, 2, 3, and Cryptic Exon 3 (CE3) with the latter resulting in an addition of 16 amino-acids to the C-terminal side of exon 3. AR-V7 mRNA can be found both in hormone naïve PCa and in CRPC [95]. AR-V7 protein is constitutively active and does not require ligand binding to translocate to the nucleus of PCa cells [95,96]. AR-V7 protein was frequently observed in CRPC specimens but rarely in hormone naïve PCa; however, when expressed in hormone naïve PCa, higher expression of AR-V7 correlated significantly with BCR following prostatectomy [95]. AR-V7 knockdown via shRNA in xenografts and CRPC cell lines caused a reduction in cell growth in an androgen-deprived environment [96].

Unlike AR-V7, AR^v567es^ expresses the very C-terminal end of the LBD, encoded by exon 8, and lacks exons 5, 6, and 7 [97]. Another variant, named AR-V12, expresses the same exons and an additional “exon 9” [97,98]. The cryptic exon 9 is also expressed in other variants such as V13, V14, V15, etc. [99]. AR^V567es^ transcript was detected in 23% of CRPC bone metastases [46,97]. Both AR-V7 and AR^v567es^ were detected in whole blood samples from CRPC patients, but only AR-V7 was detected in blood from patients who were treatment-naïve [87]. AR-V7, but not AR^v567es^, was prognostic for resistance to taxanes in CRPC patients [88]. A genetically engineered mouse model of AR^v567es^ expression resulted in epithelial hyperplasia by 16 weeks, and invasive adenocarcinoma is evident by 1 year of age [100]. Similar to AR-V7, AR^v567es^ levels were upregulated in xenograft tumors that had acquired ENZA resistance [101]. AR-V7 and AR^v567es^ homodimerize and heterodimerize with each other and also heterodimerize with full-length androgen receptor (AR-FL) in a ligand-independent manner [86].

Most known AR-Vs lack the LBD and retain the NTD. However, not all AR-Vs follow this profile—a unique 45 kDa AR variant (AR45) is detected in normal tissue that lacks the NTD and expresses the LBD preceded by a novel seven amino-acid long N-terminal extension (Figure 5). No tumorigenic function has been attributed to this variant [43]. AR45 was expressed mainly in heart and skeletal muscle, where it inhibited AR function [102]. It interacted with the NTD of full-length AR, suggesting that AR inhibition was due to the formation of AR-AR45 heterodimers. AR45 is expressed in other placental mammals as well [103] and is thought to explain increased muscle accumulation in the male phenotype [104,105]. It is therefore not expressed in PCa.

In most variants, the DBD is intact, reflecting the significant role played by this domain in AR transcriptional activity. Only one known AR-V, the cell membrane-localized and inactive AR8, splices out exon 2, thus disrupting the DBD [92,106]. Because of this unique feature, AR8 is unlikely to act as a transcription factor and is likely inactive. Different from other AR-Vs, AR8 was primarily localized on the plasma membrane, possibly through palmitoylation of two cysteine residues within the CE3 domain [106]. Nevertheless, AR8 promoted association of Src and AR with the epidermal growth factor (EGF) receptor in response to EGF treatment and enhanced tyrosine phosphorylation of AR [91,106]. Therefore, AR8 indirectly affected proliferation and apoptosis in PCa, and was required for optimal transcriptional activity of AR in response to androgens and EGF [106]. This is likely because AR8, like other AR-Vs, is co-expressed with, and dimerizes with, the full-length AR [90]. Nevertheless, the expression of AR8 remained low in patient samples [107].

## 6. AR-Targeting Strategies to Overcome ADT and ASI Resistance

**Figure 6:**
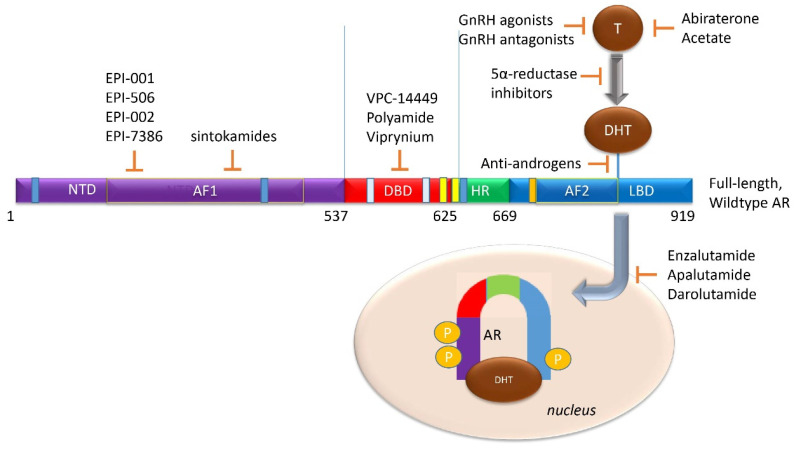
Current AR-targeted Treatments in clinical use or in development. (**LBD Targeting**) GnRH agonists (like leuprolide or goserelin), in addition to androgen biosynthesis inhibitors (like abiraterone acetate), block testosterone-mediated activation of the AR. 5-α reductase inhibitors, (like finasteride or dutasteride), and may have a role in a combination therapy to prevent testosterone conversion to its higher affinity form, DHT. Antiandrogens (like bicalutamide, nilutamide, and flutamide) are competitive inhibitors of AR ligand-binding. Direct AR inhibitors enzalutamide, apalutamide, or darolutamide, block androgen binding to the AR LBD to prevent AR activation. (**NTD and DBD Targeting**) No current clinical therapy exists to block either the AR-NTD or AR-DBD. However, several are now in development, such as the NTD-directed EPI-001 series or the DBD-targeted VPC-14449.

No clinical therapy to date completely addresses androgen-independent pro-tumor activity. The production, activity, and enrichment of AR-Vs during disease progression emphasize a clinical need for AR inhibitors that target non-LBD sites. As of 2013, all identified gain-of-function AR-Vs expressed in CRPC contain both the full NTD and DBD [59]; hence, the AR NTD and DBD remain ideal targets for inhibition and future drug therapies [93] (Figure 6).

The NTD emerges as a potential target for AR inhibition; it is implicated as a promoter of ligand-independent AR activity, has a role in nuclear localization, binds co-regulatory molecules, appears necessary for AR function in tumorigenic proliferation, and is conserved in all gain-of-function AR-Vs [9,43,85,90,108]. However, the crystal structure of the NTD has not been elucidated, making drug discovery difficult, unlike AR LBD- or DBD-inhibition where a clear ordered crystal structure has been resolved [109]. Despite this, AR NTD small molecule inhibitors have been developed. EPI-001, a mixture of four stereoisomers, blocks AR function at gene enhancer regions and was shown to bind the AR-NTD. In vivo, it prevents tumor growth in AR-expressing xenograft models with limited toxicities [108].The most potent stereoisomer, EPI-002, was developed into a prodrug, EPI-506, which was the first AR NTD inhibitor tested in a Phase 1 study in men with CRPC. The drug was well-tolerated but required high doses (>1280 mg) to achieve minor and transient PSA declines, reflecting EPI-506′s low potency and short half-life [110,111]. A newer molecule, EPI-7386, synthesized from EPI-002 demonstrates >20-fold improved potency and higher stability [112] (Figure 6).

Beyond EPI-001 and derivative compounds, NTD targeting therapies have been tested. Chlorinated peptides, called sintokamides, extracted from a marine sponge, were shown to target the AR-NTD. Sintokamide A inhibited LNCaP proliferation while reducing AR transcriptional activity [113,114,115]. Additionally, decoy peptides with the AR-NTD sequence of AR have been shown to reduce PCa tumor development and serum PSA levels [116]. These peptides may reduce AR activity by interacting with other proteins needed for normal AR activation.

The DBD is an easier domain to target based on rational design as docking programs have the ability to identify candidate inhibitors and improve such inhibitors for specificity [117]. The DBD persists in all gain-of-function AR-Vs (along with the NTD), linking both full-length and truncated AR homo- and heterodimers to AREs [14]. DNA-dependent dimerization of the full-length AR requires the DBD [83], as does AR-V androgen-independent nuclear localization [14]. The DBD is critical for ligand-independent AR activity [118]. Thus, DBD inhibitors can be designed to potentially abolish AR transcription by prevention of AR dimerization, the direct blocking of full-length AR and AR-V binding to DNA at ARE sites, or the inhibition of AR-V localization to the nucleus. However, DBD inhibition should be done with caution, as the AR DBD shares sequence homology with the glucocorticoid and estrogen receptors [117], and accidental inhibition of these domains may lead to undesired side effects.

A surface-exposed pocket on the AR-DBD was identified as an alternative drug-target site for AR inhibition, and small molecules designed to selectively bind the pocket effectively block transcriptional activity of full-length AR and AR-V forms at low- to sub-micromolar concentrations [117]. The compounds did not impede nuclear localization of the AR, but they blocked interactions with chromatin and demonstrated the inhibition of gene expression and tumor volume in mouse xenografts [117]. One of these compounds, named VPC-14449, showed limited interference to the structurally homologous glucocorticoid and estrogen receptor DBDs, and reduced both tumor volume and PSA serum levels [117]. However, the structure of this compound was later found to be erroneous and was corrected [117] (Figure 6).

Another way to target the DBD is by using a polyamide designed to bind to the consensus sequence of ARE sites and disrupt AR binding [116]. The main goal is to reduce gene expression of AR targeting genes as resistance to normal AR inhibition is common. However, polyamides can be created to target specific DNA sequences and act like normal DNA binding proteins, bypassing the problem of inhibiting AR splice forms, mutations, and other forms of resistance. When tested, polyamides were able to bind to the ARE sequences and reduce gene expression of KLK3/PSA, KLK2, FKBP5, and TMPRSS2. Although one of the polyamides reduced PSA expression as efficiently as bicalutamide, overall, they were unable to affect other AR targeted genes as single agents, which limits their clinical use.

Salts of pyrvinium (Viprynium), an anti-helmintic therapeutic effective against pinworms, was shown to inhibit the growth of cancer cells; in particular, this inhibition of growth occurred during glucose starvation [119]. It was found that pyrvinium inhibits AR-dependent gene expression in the prostate gland in vivo and induces prostate atrophy [120]. Pyrviniumpamoate (PP) is a potent noncompetitive inhibitor of the AR in PCa which inhibits activity of the AR-Vs via binding at the interface of the DBD dimer and the minor groove of the ARE [121]. PP also inhibits ligand-independent AR activation by HER2 and inhibited the in vivo growth of CRPC xenografts that express AR-V. However, although PP decreased prostate weight and did not affect lean body mass, it also decreased bone mineral density [121]. Later studies showed that several splicing factors, such as DDX17, had reduced interactions with AR in the presence of pyrvinium [122]. However, pyrvinium did not alter the levels of AR-Vs in several PCa cell lines [121,122]. As a result, this drug, though promising, has not progressed to the stage of clinical investigation. 

Other anti-helminthic drugs that target the AR-Vs have been identified, including niclosamide [123]. This drug targeted the IL6-Stat3-AR pathway to overcome ENZA resistance and inhibited migration and invasion in CRPC [124]. In addition, niclosamide re-sensitized resistant cells to treatment with ABI and ENZA in vitro and in vivo [125,126]. The original niclosamide formulation was tried in a clinical study in men with CRPC but showed limited efficacy due to lack of plasma concentrations above the clinically significant threshold [127]. Several clinical trials are currently undergoing with a newly formulated niclosamide plus abiraterone acetate (NCT02807805), and niclosamide plus enzalutamide (NCT03123978) in men with CRPC. Although there was no investigation as to whether niclosamide binds to the AR DBD, based on the similarities to PP, this seems to be a possible mechanism. These compounds emphasize the merits of using small molecules to inhibit AR alternative sites.

## 7. Conclusions

In summary, NTD and DBD are attractive targets for AR inhibition. Such inhibitors may extend survival and improve quality of life, especially when ADT or current ASIs fail. However, predictable challenges will arise due to the highly mutagenic nature of cancer. The NTD and DBD, like LBD, may acquire point mutations [9], potentially rendering small molecules ineffective as AR inhibitors. Even worse, as in LNCaP and LNCaP-derived cell lines, the AR may mutate to elicit agonistic effects from small molecule inhibitors. For example, the T877A point mutation in the LBD of these lines allows the inhibitor bicalutamide to promote, instead of inhibit, AR genomic activity [21]; similar effects may be seen in the AR DBD-targeting drugs as well. Finally, these inhibition strategies will not work in PCa that no longer rely on the AR for disease progression. Unfortunately, such targeting strategies may even select and enrich for cancerous cells that act independently of AR. Nonetheless, the potential benefits of alternative inhibition sites to overcome numerous mechanisms of resistance that render AR LBD direct and indirect inhibitors ineffective may ultimately extend survival and improve quality of life for those diagnosed with PCa.
